# Soluble ST2 as a Potential Biomarker in Pericardial Fluid of Coronary Artery Patients

**DOI:** 10.21470/1678-9741-2020-0317

**Published:** 2021

**Authors:** Reşat Dikme, Mahmut Padak, Mesut Işık, İsmail Koyuncu, Ebru Temiz, Mehmet Salih Aydın, Ömer Göç

**Affiliations:** 1 Department of Perfusion Techniques Program, Vocational School of Health Services, Harran University, Şanlıurfa, Turkey.; 2 Department of Bioengineering, Faculty of Engineering, Bilecik Seyh Edebali University, Bilecik, Turkey; 3 Department of Medical Biochemistry, Faculty of Medicine, Harran University, Şanlıurfa, Turkey.; 4 Department of Medical Biology and Genetics, Vocational School of Health Services, Harran University, Şanlıurfa, Turkey.; 5 Department of Cardiovascular Surgery, Faculty of Medicine, Harran University, Şanlıurfa, Turkey.

**Keywords:** Coronary Artery Disease, Pericardial Fluid, Real-Time Polymerase Chain Reaction, IL1RL1 protein, human, Biomarkers, Prognosis

## Abstract

**Introduction:**

The growth Stimulation expressed gene 2 (ST2) (or interleukin 1 receptor-like 1, also known as IL1RL1) is considered a biomarker of poor prognosis in cardiovascular diseases. The aims of this study are to investigate ST2 in the pericardial fluid (PF) of coronary artery disease patients and to contribute to the understanding of the pathophysiology of coronary artery disease.

**Methods:**

40 patients (blood plasma and PF) who underwent coronary artery bypass surgery and 40 controls (blood plasma only) were included in this study. Soluble ST2 (sST2) level was determined by enzyme-linked ımmunosorbent assay method in plasma and PF, and sST2 gene expression was determined by quantitative real-time polymerase chain reaction (QRT-PCR) method.

**Results:**

The sST2 level was found to be 44.89 ng/ml and 390.357 ng/ml in the control and patient groups’ plasma, and 223.992 ng/ml in the PF of the patient group. An increase in sST2 level was detected in the patient group compared to the control group (*P*<0.001). The sST2 expression in plasma was higher in the patient group than in the control group. Additionally, sST2 was more expressed in the plasma of the patient group than PF (*P*<0.001).

**Conclusion:**

The fact that sST2 was detected for the first time in a high level in PF showed that this biomarker was closely related with the heart and strengthened its potential to be used as a biomarker. Therefore, sST2 can contribute to the understanding of the pathophysiology of coronary artery disease.

**Table:** 

Abbreviations, acronyms & symbols				
**AVG**	**= Average**		**mRNA**	**= Messenger ribonucleic acid**
**CAD**	**= Coronary artery disease**		**PCR**	**= Polymerase chain reaction**
**cDNA**	**= Complementary deoxyribonucleic acid**		**PF**	**= Pericardial fluid**
**CPB**	**= Cardiopulmonary bypass**		**PHFS**	**= Penn Heart Failure Study**
**Ct**	**= Cycle threshold**		**RNA**	**= Ribonucleic acid**
**CVD**	**= Cardiovascular diseases**		**QRT-PCR**	**= Quantitative real-time polymerase chain reaction**
**DNA**	**= Deoxyribonucleic acid**		**sST2**	**= Soluble growth Stimulation expressed gene 2**
**ELISA**	**= Enzyme-linked immunosorbent assay**		**ST2**	**= Growth Stimulation expressed gene 2**
**FHS**	**= Framingham Heart Study**		**ST2L**	**= Membrane-bound growth Stimulation expressed gene 2 or mST2**
**GE**	**= Genomic elution**			
**IL33**	**= Interleukin 33**		**STEMI**	**= ST-elevation myocardial ınfarction**

## INTRODUCTION

The incidence of cardiovascular diseases (CVD), which is one of the most important causes of mortality and morbidity in the world, is rapidly increasing. Despite the ongoing developments in their treatment, these diseases are still in the first place as the cause of death in the People’s Republic of China^[1]^.

**Fig. 1:**
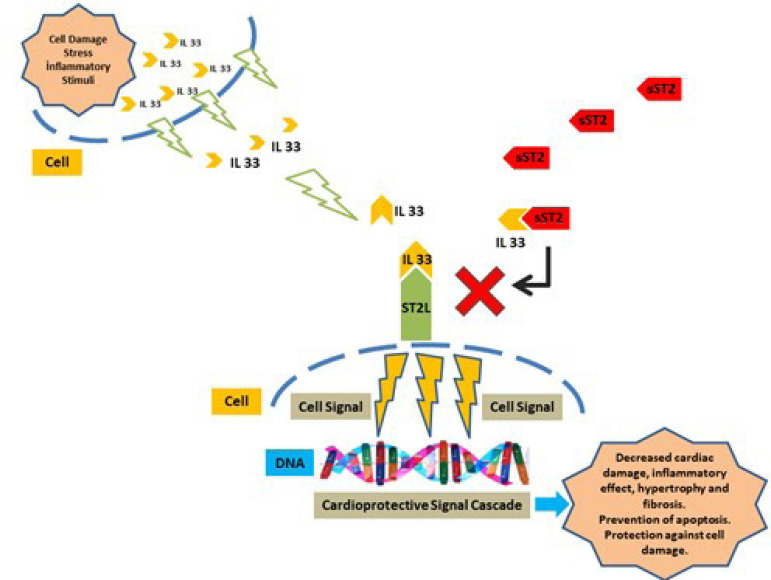
Cardioprotective relationship of interleukin 33 (IL33) with membrane-bound growth STimulation expressed gene 2 (ST2L) and blocking with soluble growth STimulation expressed gene 2 (sST2). DNA=deoxyribonucleic acid.

A biomarker expressed in the heart tissue and coronary endothelial cells can be released into the blood and pericardial fluid (PF). Since some of the heart-related biomarkers detected in the circulation are expressed in both the heart muscle and skeletal muscle, analyses in the blood may not indicate heart disease. Therefore, besides blood, PF can be used for diagnosis in CVD. The PF within the pericardial cavity, which is the double-walled pouch enveloping the entire heart, shows dynamic changes. The pericardium has immunological, paracrine, vasomotor, and fibrinolytic activity and it affects the myocyte structure, function, and gene expression by synthesizing biomolecules such as eicosanoids (prostaglandin, thromboxane, and leukotriene)^[2]^. Recently, the growth Stimulation expressed gene 2 (ST2) (or ınterleukin 1 receptor-like 1 [also known as IL1RL1], T1, Fit1, IL33R, DER4) has been considered as an important prognostic biomarker because of its involvement in the pathogenesis of CVD, its stability, and easy measurement in clinical samples. ST2, which plays an important role in cellular stress and inflammatory response, is the receptor of ınterleukin 33 (IL33)^[3]^. IL33, with its cytokine and transcriptional feature, creates a signal between cells as a nuclear factor and helps to protect the heart^[4,5]^. IL33, which creates a paracrine effect between cardiac myocytes and fibroblasts when there is mechanical load in the heart, acts by binding to ST2^[4]^. ST2 has two important isoforms, these isoforms are membrane-bound ST2 (ST2L or mST2) and soluble ST2 (sST2). ST2L is not free in peripheral circulation. sST2 without transmembrane and intracellular domain moves freely in the peripheral circulation^[5]^. While binding of ST2L to IL33 has a cardioprotective effect (mechanical stimulation, decreased cardiac damage, prevention of apoptosis, lowered inflammatory effect, hypertrophy, and fibrosis), binding of sST2 to IL33 causes these positive effects to disappear (Figure 1). sST2 acts as a trap receptor (decoy receptor), reducing the positive effects of IL33^[4]^.

High level of sST2 indicates that the heart is under intense stress. This causes cellular death, tissue fibrosis, decreased heart function, and increased rate of disease progression. Therefore, sST2 is considered to be a biomarker of poor prognosis in CVD^[5]^. It was found that sST2 rising above the normal level increased many negative conditions due to CVD by approximately three times^[6]^. According to multiple clinical studies, sST2 has emerged as a clinically useful prognostic biomarker in patients with CVD, such as myocardial infarction, acute dyspnea, and heart failure, and in the low-risk population^[7]^.

The main source of sST2 in individuals with healthy and different diseases is currently not fully determined. In a study of neonatal rat heart myocytes related to heart disease, the sST2 in the bloodstream was found to be of myocardial origin^[8]^. However, some studies have shown that the predominant source of sST2 in cardiac patients may be vascular endothelial cells rather than the myocardium^[9]^. When all the studies are evaluated together, it is revealed that sST2 is produced by cardiac fibroblast, cardiomyocytes, macrovascular (aorta and coronary arteries), and microvascular endothelial cells in case of damage and stress. In another study, parallel increased release of both ST2L and sST2 was detected in cardiomyocytes and cardiac fibroblasts under biomechanical stress^[10]^. However, there are no studies on sST2 release in epicardium and pericardium. We assume that two of the sources of sST2 in humans are probably epicardium and pericardium. In coronary artery disease (CAD) patients, research of sST2 only in the blood is not sufficient. For this reason, it is also important to detect sST2 in the PF, the tissue closest to the heart. The fact is that the increase mechanism of ST2, which has a negative effect on the formation of CAD, is not known; there is no ST2 study in the PF in the literature, and unanswered questions about the IL33-ST2 pathway make this study important. Enzyme-linked ımmunosorbent assay (ELISA) kits are often used to measure human sST2 levels in serum and plasma. In this study, differently, gene expression study was performed for the measurement of sST2.

## METHODS

### Patients Included in the Study

The study protocol was carried out in accordance with the Helsinki Declaration as revised in 1989. Signed informed consent forms were obtained from the families of all participants. Ethical approval was obtained from the Harran University Local Research Ethics Committee (Ethical approval number: 05.01.2017-17/01/23). Forty patients who underwent coronary artery bypass surgery with cardiopulmonary bypass (CPB) method were included in the study. Both PF and blood were taken from the patient group. As for the control group, only blood samples were taken from 40 healthy individuals in the same age group.

### Obtainment of PF

The pericardium was opened after median sternotomy was performed in patients undergoing coronary bypass surgery with the CPB method. Then, PF was aspirated with a sterile syringe. The aspirated PF was placed in sterile tubes without gel (10 ml, Vacutainers/BD Biosciences) and put in an ice-filled container. Then, the PF in the sterile tube was purified from the cells by passing through the centrifugation step at 5000 rpm for five minutes. After the centrifugation step, the supernatant was taken to a ribonuclease-free tube (1.5 mL, Eppendorf) and stored at -80 °C.

### Obtainment of Blood Plasma

Blood was taken from the patients who underwent coronary bypass surgery with the CPB method, before CPB, and gel-free anticoagulant (0.2 ml, Nevparin injectable 25000 IU/5 mL) was placed in sterile glass tubes (10 ml, Vacutainers/BD Biosciences). These tubes were immediately placed in an ice-filled container and delivered to the laboratory. Then, sterile tubes were centrifuged at 5000 rpm for five minutes. After the centrifugation step, plasma was taken into a ribonuclease-free tube (1.5 mL, Eppendorf) and stored at -80 °C. After collecting PF and blood from 40 patients, ELISA and quantitative real-time polymerase chain reaction (QRT-PCR) studies were performed.

### Studying Stages of ST2 Gene Expression in PF and Plasma

#### 1. Total ribonucleic acid (RNA) (messenger RNA [mRNA]) Isolation in PF and Plasma

For isolation of mRNA in PF and plasma, Qiagen (Qiagen GmbH, Hilden, Germany) miRNeasy Serum/Plasma Kit (Cat No./ID: 217184) was used. The working procedure of the manufacturer company was followed and total RNA isolation was performed.

#### 2. Complementary deoxyribonucleic acid (cDNA) Obtainment from mRNA in PF and Plasma

In order to transfer mRNA in obtained total RNA to cDNA, RT2 HT First Strand Kit (Qiagen, GmbH, Hilden, Germany, Cat No./ID: 330411) was used. Each reverse transcribe phase was carried out twice. The contents of reverse transcriptase PCR reaction mixture applied to obtain cDNA from mRNA were prepared as in Table 1. According to the work flow chart of the kit, the relevant procedure was followed.

**Table 1:** Reverse transcriptase PCR mixture for obtaining cDNA from mRNA.

Ingredients	Amount
RNA	500 ng/µl
Buffer GE (removal of genomic DNA)	2 µl
Ribonuclease-free water	Adjusted to total 10µl

cDNA=complementary deoxyribonucleic acid; DNA=deoxyribonucleic acid; mRNA=messenger ribonucleic acid; GE= Genomic elution; PCR=polymerase chain reaction; RNA=ribonucleic acid

#### 3. Quality and quantity identification from cDNA obtained from PF and plasma

Quality and quantity identification was done by using Thermo Scientific Multiskan Spectrophotometer System (Waltham, Massachusetts, Unite States of America) equipment. Before expression analysis experiments, cDNA quantities obtained from samples were declined to 50 ng/µl for mRNA cDNA.

#### 4. Real-time PCR (QRT-PCR) Method

Before QRT-PCR, mRNA-cDNA preamplification was performed in PF and plasma, and then routine PCR protocol was applied. Rotor Gene 6000 Real-Time PCR Machine (Qiagen GmbH, Hilden, Germany) device and QuantiTect SYBR Green PCR Kit (Qiagen GmbH, Hilden, Germany, Cat No./ID: 204143) were used for QRT-PCR.

Primer pool: 40 µl (20 µl F [10 pmol/µl] + 20 µl R [10 pmol/µl]) primer was used; 210 µl of water was added per primer and the relevant content is given in Table 2.

**Table 2:** Primary pool mix for ST2 gene expression.

For 1 sample	
5´ miScript master mix	12.5 µl
Taq polimeraz	0.5 µl (250 unit)
Primer pool	7.5 pmol/µl
**Total mix: 20.5µl**	

ST2=growth Stimulation expressed gene 2

The reaction was carried out at 25.5 µl by adding 5 µl cDNA to the total mix. Routine PCR procedure was applied according to Table 3 for sST2 expression level in PF and Plasma.

**Table 3:** Routine PCR protocol for ST2 expression level.

Temperature (^o^C)	Time	
95	10 minutes	
94	15 seconds	}12 times
60	2 minutes	
4		

PCR=polymerase chain reaction; ST2=growth STimulation expressed gene 2

The product obtained after PCR was diluted with 91 µl of water; 5 µl was used for real time PCR (QRT-PCR). For QRT-PCR, the component and volume were used according to Table 4.

**Table 4:** QRT-PCR components for sST2 expression level.

Components	Volume
RT^2^ SYBR^®^ Green qPCR Mastermix	5 µl
F Primer (10 pmol/µl)	0.3 µl
R Primer (10 pmol/µl)	0.3 µl
Ribonuclease/deoxyribonuclease-free water	3.9 µl
cDNA (50 ng/µl)	0,5 µl
**Total**	10 µl

cDNA=complementary deoxyribonucleic acid; QRT-PCR=quantitative real-time polymerase chain reaction; sST2=soluble growth STimulation expressed gene 2

The prepared mixture was distributed to the tubes to be 9.5 µl; 0.5 µl cDNA of each sample was added to each tube containing the QRT-PCR premix and the study was performed under the PCR conditions set forth in Table 5.

**Table 5:** QRT-PCR settings for ST2.

	Temperature (ºC)	Time	
Enzyme activation	95	10 minutes	
Denaturation	95	15 seconds	}40 times
Primer binding/elongation	60	60 seconds	

QRT-PCR=quantitative real-time polymerase chain reaction; ST2=growth STimulation expressed gene 2

#### 5. Primer Design

In this study, ST2 primers (Metabion International AG/metabion GmbH, Germany) and beta-actin, a housekeeping gene, were used. ST2 primers were designed using the "Gene" interface (http://www.ncbi.nlm.nih.gov/tools/primerblast/) in the National Center for Biotechnology Information database. The sequences of the ST2 and beta-actin primers are shown in Table 6.

**Table 6:** ST2 and beta-actin forward and reverse primers.

	Forward primer	Reverse primer
ST2	5'-TTCCAGTAATCGGAGCCCCT-3'	5'-AACCATGTGCAAGGGGAAGAG-3'
Beta-actin	5'-CGTACCACAGGCATTGTGATG-3'	5'-TTTGATGTCACGCACGATTTC-3'

ST2=growth STimulation expressed gene 2

### ST2 ELISA Study in PF and Plasma

Human sST2 ELISA Kit (Elabscience Biotechnology Co., Ltd., Product ID: E-EL-H1615) was used to determine the ST2 level in PF and plasma by ELISA method. Samples in PF and plasma were kept at room temperature for two hours before working. Then, sST2 levels were measured according to the manufacturer's operating procedure.

### Statistical Analysis

After the studies were completed, it was checked whether the data were normally distributed. Then, one-way analysis of variance was performed. According to the Shapiro-Wilk test result, Student's *t*-test was used for the significant difference test in the normal distribution of the groups, and the Mann-Whitney U test was used in the abnormal distribution. In our study, Mann-Whitney U test was used for statistical analysis of sST2 ELISA results in PF and plasma, and Student *t*-test was used in sST2 gene expression results. By comparing each test group with the control groups, *P*-values <0.05 were considered significant. In our study, “prism 6” program (GraphPad Software, Inc, San Diego, United States of America) and QIAGEN analysis program (https://www.qiagen.com/us/shop/genes-and-pathways/data-analysis-center-overview-page/) were used for statistical analysis.

## RESULTS

**Fig. 2:**
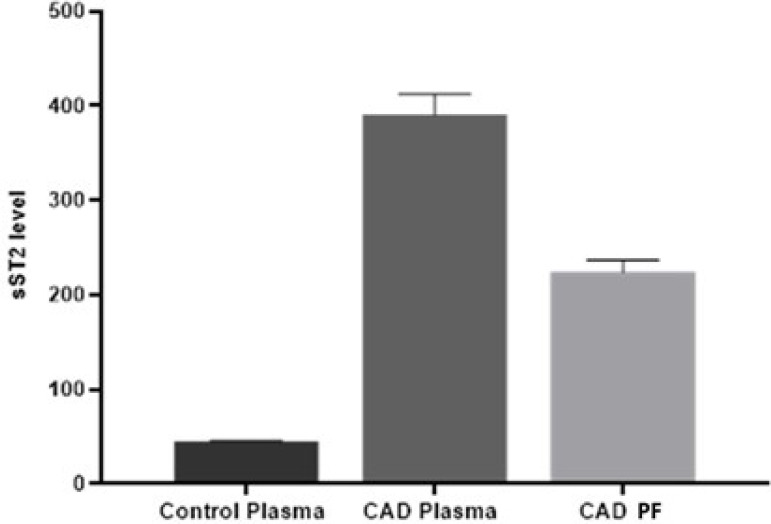
Determination of soluble growth STimulation expressed gene 2 (sST2) level of as a biomarker for coronary artery disease (CAD) in pericardial fluid (PF) and plasma.

**Fig. 3:**
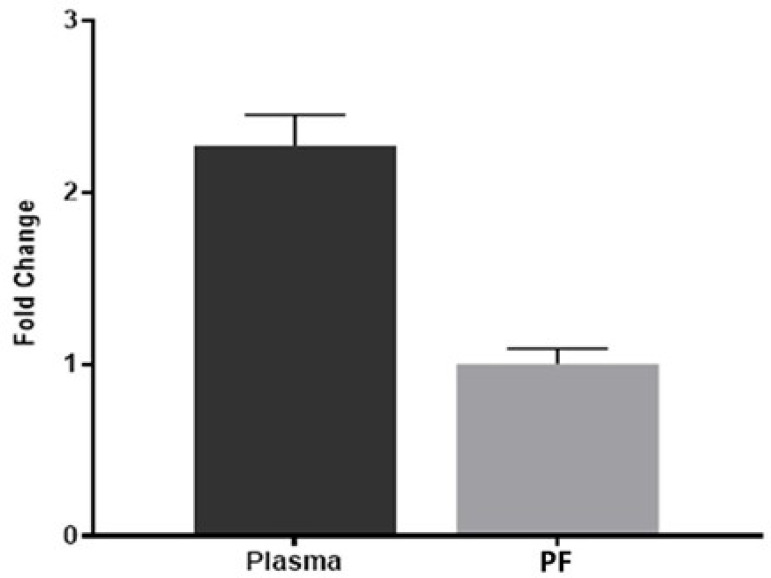
Fold change values of soluble growth STimulation expressed gene 2 in pericardial fluid (PF) and plasma of coronary artery disease.

As a result of our ELISA study, the sST2 level was found to be 223,992±19,628 ng/ml in PF, and 44,89±4,226 ng/ml and 390,357±25,245 ng/ml in plasma of the control and patient groups (*P*<0.001) (Figure 2). In our study, the fold changes of sST2 gene expression levels in PF and plasma were evaluated. The fold changes of sST2 gene expression levels in PF and plasma were evaluated with Student’s *t*-test. As a result, the average Ct value for sST2 was found as 13.33 in PF, and 19.69 and 2.26 in the plasma of the patient and control groups, respectively. As a result, the average Delta (Ct)* value for sST2 was found as 0,790741 in PF, and 0,39037 and -0,7450 in the plasma of the patient and control groups, respectively. While 22^^(- Ort.Delta CT)^ value of sST2 was found as 0.578047 in PF, it was found as 1.31073 and 0.1506 in the patient and control groups’ plasma, respectively. The sST2 fold change (2^-ΔΔCt^) in the plasma of patients was calculated as 2.2675 compared to PF (Figure 3). As a result of statistical analysis, significant changes were found in the replicate 2^^(- Delta Ct^) values of sST2 gene expression in PF and plasma (*P*<0.001).

## DISCUSSION

In the American College of Cardiology Foundation/American Heart Association - ACCF/AHA - guide, ST2 was defined as a “novel biomarker” in 2013^[11]^. The sST2, a biomarker independent of natriuretic peptides and cardiac troponins, has been accepted as an important biomarker in many diseases such as ST-elevation myocardial ınfarction (STEMI), non-STEMI (or NSTEMI), CAD, heart failure, valvular diseases, cardiomyopathy, coronary bypass, acute cardiac allograft rejection, and acute Kawasaki disease^[12]^. According to another study, sST2 shows itself as a strong ischemic marker and is used especially in predicting the etiologies of cardiomyopathy^[11]^. Studies have reported that ST2 is not affected by factors such as age, body mass index, and renal dysfunction, and its *in vitro* stability is high^[13,14]^.

According to the Framingham Heart Study (FHS) Cohort analysis, the sST2 level gives important information in CAD asymptomatic individuals^[15]^. The most important reference values for circulating sST2 were obtained from the FHS. While the reference range of ST2 level was 11-45 ng/mL in men (n=462), sST2 was found to be 9-35 ng/mL in women (n=674)^[15,6]^.

Dieplinger et al.^[6]^, while the normal mean value of sST2 was 18 ng/ml, stated that concentrations > 35 ng/ml were at high risk for CVD. In the Penn Heart Failure Study (PHFS), it was stated that high sST2 levels gave a predictive idea about death and heart transplantation in patients with heart failure^[16]^. According to the PHFS, patients with sST2 value > 35 ng/ml were found to have 2.8 times more risk of CVD than normal individuals^[17]^.

Another study reported that heart patients with high sST2 values may have an average of 32% heart failure and 45% death after 11 years^[7]^. The increase in the level of sST2 in patients with stable CAD may have been caused by chronic inflammation in atherosclerosis. According to large-scale cohort analysis, sST2 was determined as an independent biomarker for cardiovascular mortality in stable CAD patients. According to a study, long-term mortality has been estimated in stable CAD patients with a sST2 value of 24.6 ng/mL in blood plasma, and it has been reported that the risk of death increases two-fold in people above this value^[18]^.

According to the researches, only ST2 determined in plasma is insufficient for the correct diagnosis of the disease. The fact that the ST2 increasing mechanism that causes the formation of CAD is unknown and the absence of sST2 and gene expression studies in PF indicate that this study is important. In this study, it was aimed to determine ST2 and its gene expression level in PF of cardiovascular patients. The demographic characteristics of the patients included in the study for this purpose are presented in Table 7.

**Table 7:** Demographic characteristics of the control and patient groups included in the study.

Variable	Control	Patients
Gender (female/male, %)	12/28 (30%/70%)	13/27 (32.5%/67.5%)
Age (years)	63	61

As a result of our study, compared to the control group, sST2 level of CAD patients was found to be 8.5 times higher in plasma and 5.5 times in PF.

The sST2 expression results were analyzed statistically both in the PF and plasma of the patient group and in the plasma of the control group. The statistical results of the mean average (AVG) cycle threshold (Ct), AVG Delta Ct, 2^^(-Avg.(Delta(Ct))^, fold change, and *P*-values obtained from sST2 are presented in Table 8. As a result of statistical analysis, significant changes were found in the replicate 2^^(- Delta Ct^) values of sST2 gene expression in PF and plasma (*P*<0.001).

**Table 8:** Statistical results of gene expression of sST2 in PF and plasma.

	Average Ct			Average Delta (Ct)			2^^(-AVG.(Delta(Ct))^			Fold change	*P*-value
sST2	PF	CAD plasma	Control plasma	PF	CAD plasma	Control plasma	PF	CAD plasma	Control plasma		
	13.33±0.59	19.69±2.88	2.26±0.34	0.790741	0.39037	0.7450	0.57805	131.073	0.1506	22675	**0.000265**

AVG Delta (Ct)=(Ct (GOI) - AVG Ct (HKG)). AVG=average; CAD=coronary artery disease; PF=pericardial fluid; Ct=cycle threshold; sST2=soluble growth STimulation expressed gene 2

In our study, the increase in sST2 gene expression and sST2 level in CAD plasma and PF supports the prediction that sST2 value > 35 ng/ml increases the risk of CVD. This is actually an expected situation. In previous studies, it was noted that increased sST2 level, which inhibits the cardioprotective effect of IL33, is a biomarker in the diagnosis of CVD. High level of sST2 in the patient group may play an active role in the progression of the disease by contributing to the pathophysiologic mechanism in patients with CAD. While there are many tissues that increase the circulating level of sST2, the most important factor that increases the level of sST2 in PF is cardiovascular tissues, because sST2 release to PF can only be from the closest heart tissue.

In many previous studies, although sST2 release is known to increase in heart and lung diseases, its release focus and rate are still unknown^[19]^. There are studies showing that sST2 protein is secreted after vascular endothelial cells IL-1b, tumor necrosis factor, and phorbol ester stimulation in response to an inflammatory stimulus^[20]^. In some studies, it was found that there is an increase in sST2 in plasma of patients with pulmonary artery hypertension^[21]^. In another study, ıt was reported that human peripheral blood cells and mast cells derived from mouse bone marrow and stimulated subsequently produce soluble sST2^[22]^.

### Limitations

Our study has some limitations. This is a retrospective non-randomized study and it included a limited number of patients. Since PF cannot be obtained from healthy people, ST2 analysis could not be performed on them. Our main interest was to investigate the prognostic value of ST2 in this study. Differently designed studies about the pathophysiological processes are needed to elucidate the underlying mechanisms of our findings.

## CONCLUSION

In conclusion, determining the sST2 level only in plasma may not give very clear results for the reasons mentioned. Therefore, in order to make a real prediction, determining sST2 in both plasma and PF is important for knowing the oscillation focus. The study is consistent with previous studies showing that the main source of increased sST2 is not only the myocardium. Before our study, it was assumed that one of the sST2 sources in humans is probably the pericardium. In our study, the detection of high amounts of sST2 levels and gene expression in the PF of the patient group confirmed this prediction. In addition, the increase in both the level of sST2 gene expression and its amount in PF supports the prediction that it can be used as a prognostic biomarker for heart disease. Knowing the sST2 release foci can contribute to the development of more effective treatment methods, such as suppressing sST2 expression with various treatments or preventing sST2-IL33 binding.

**Table:** 

Authors' roles & responsibilities	
RD	Substantial contributions to the conception or design of the work; or the acquisition, analysis, or interpretation of data for the work; final approval of the version to be published
MP	Substantial contributions to the conception or design of the work; or the acquisition, analysis, or interpretation of data for the work; final approval of the version to be published
MI	Substantial contributions to the conception or design of the work; or the acquisition, analysis, or interpretation of data for the work; final approval of the version to be published
IK	Substantial contributions to the conception or design of the work; or the acquisition, analysis, or interpretation of data for the work; final approval of the version to be published
ET	Substantial contributions to the conception or design of the work; or the acquisition, analysis, or interpretation of data for the work; final approval of the version to be published
MSA	Substantial contributions to the conception or design of the work; or the acquisition, analysis, or interpretation of data for the work; final approval of the version to be published
ÖG	Substantial contributions to the conception or design of the work; or the acquisition, analysis, or interpretation of data for the work; final approval of the version to be published
